# Understanding Sexual Complaints and History Taking: A Standardized Patient Case on Dyspareunia for Obstetrics and Gynecology Clerkship Students

**DOI:** 10.15766/mep_2374-8265.11001

**Published:** 2020-10-29

**Authors:** Jill M. Hagey, Jordan Toole, Kelly Branford, Tracey Reynolds, Elizabeth Livingston, Sarah K. Dotters-Katz

**Affiliations:** 1 Resident Physician, Department of Obstetrics and Gynecology, Duke University Medical Center; 2 Undergraduate Medical Education Coordinator, Department of Obstetrics and Gynecology, Duke University Medical Center; 3 Director of Clinical Skills Program, Office of Curricular Affairs, Duke University School of Medicine; 4 Standardized Patient Coordinator, Office of Curricular Affairs, Duke University School of Medicine; 5 Professor, Department of Obstetrics and Gynecology, Duke University Medical Center; 6 Assistant Professor, Department of Obstetrics and Gynecology, Duke University Medical Center

**Keywords:** Standardized Patient, Curriculum Development, OB/GYN, Clinical Skills Assessment/OSCEs

## Abstract

**Introduction:**

Learning to elicit a sexual history and counsel patients on sexual pain aligns with the Association of Professors of Gynecology and Obstetrics clerkship objectives. This topic can be challenging to cover due to lack of exposure in clinical encounters and inadequate coverage in preclinical studies.

**Methods:**

Second-year medical students in the OB/GYN clerkship participated in a standardized patient (SP) encounter on dyspareunia, receiving formative feedback on sexual history taking, differential diagnosis and management plan, and their SP's comfort during the encounter. Student feedback was obtained mid- and postclerkship. Summary statistics and regression models comparing SP encounter scores with shelf exam and clerkship scores are reported.

**Results:**

From September 2018 through July 2019, 101 students completed the encounter. Students asked an average of 3.9 of 13 sexual history questions. Sixty-six percent of students identified a correct diagnosis; 48% provided a management plan. The majority of students were very good or excellent at creating a safe environment. Most reported the encounter enhanced their learning (62%) and identified knowledge gaps (72%). SP encounter score was not associated with shelf exam score or clerkship letter grade but was associated with final clerkship score (unadjusted ß = 0.2, *p* = .009; adjusted ß = 0.1, *p* = .2). A summary didactic session was developed following student feedback.

**Discussion:**

This SP encounter and summary didactic session allowed students to improve their sexual history taking and may be associated with clerkship performance. These skills are necessary to advocate for patients with sensitive complaints across specialties.

## Educational Objectives

By the end of this activity, learners will be able to:

1. List three to five diagnoses that can lead to dyspareunia and explain the defining features of each diagnosis.
2. Use verbal and nonverbal cues to create a comfortable environment for a standardized patient to share information about her sexual history and sexual concerns.
3. Engage a standardized patient in discussion of treatment options for dyspareunia, including responding to specific concerns voiced by the patient.

## Introduction

Sexual pain and dyspareunia are common sexual complaints, with 8%-21% of women globally reporting symptoms.^1^ Understanding these concerns and knowing how to take a sexual history are important components of the medical student curriculum in the obstetrics and gynecology clerkship, as laid out by the Association of Professors of Gynecology and Obstetrics (APGO).^2^ Despite this objective, the majority of medical school students feel ill prepared to take a sexual history and address clinical sexual concerns in the future careers.^3^ Additionally, within our university, sexual pain and dyspareunia have been identified as orphan topics within the medical school curriculum that are not adequately covered in either clinical experiences or didactic teaching.

While there is little high-quality data regarding how to teach sexual history taking and respond to sexual complaints, practice patient interviews with one-on-one feedback may help students feel more comfortable with these skills.^4^ Indeed, within a comprehensive sexual history curriculum for medical students, history taking with patients and directed feedback were rated by students as the most effective aspect of the curriculum and increased their likelihood of taking a sexual history in the future.^5^ Standardized patient (SP) vignettes can be used to teach a variety of topics and to evaluate competencies such as interpersonal communication and professional values.^6^ Communication skills appear to be the strongest predictor for sexual history taking among practicing physicians.^7^ In addition to improving specific clinical skills, SP encounters and other simulation exercises have also been shown to increase clinical knowledge based on written tests and performance of skills across other specialties.^8–10^ Other interventions to teach sexual history taking include lectures and small-group sessions, through both interactive workshops and didactic presentations.^4,5^

While other SP cases have been described as teaching tools, we were unable to identify any formal SP curricula specifically focused on dyspareunia. A systematic review of educational programs on sexual history taking identified 11 educational programs implemented among medical students, residents, and practicing physicians to improve sexual history taking skills.^4^ Most of these interventions included didactics or training sessions without an interactive portion for physicians or trainees to practice their skills. For those interventions that included small-group practice sessions or SP encounters, improved skill in taking a sexual history and counseling on sexual behavior was noted.^10–14^ However, no specific information on these practice sessions was included, limiting the ability for widespread utilization of these tools. A communications workshop on sexual history taking for medical students with specific tools for use was found, but it has often been used without opportunity for skills practice among students.^15^ Furthermore, a search of PubMed for the topics dyspareunia and pelvic pain related to SPs and medical education did not generate any specific teaching tools available for use.

We describe the creation and implementation of an SP case and complementary summary didactic session on dyspareunia as a teaching session for formative feedback for obstetrics and gynecology clerkship students at the Duke University School of Medicine.

## Methods

### Target Audience

Medical students participated in a 6-week obstetrics and gynecology clerkship as part of their core clinical rotations during their second year at the Duke University School of Medicine. All learners in the obstetrics and gynecology clerkship participated in subrotations of 2 weeks each in an obstetrics experience, gynecology experience, and outpatient experience. Students received three lectures on obstetrics and gynecology topics during their first year of medical school prior to coming to their clerkship, as well as introductory training on communication and counseling of patients. Students had no formalized curriculum regarding dyspareunia prior to the encounter, but they were encouraged to study throughout the obstetrics and gynecology clerkship based on the APGO medical student objectives.

Students in the clerkship had didactic teaching sessions weekly during their clerkship, and we held the SP encounter during the third week of those didactic sessions. Students had completed one full subrotation prior to undergoing the SP case. We instructed students that they would perform two cases with SPs in our simulation center during their didactic time as part of a clinical performance encounter. Eight students completed these cases at a time, spending a total of 1 hour in the simulation center divided into two 30-minute cases. All students participating in the clerkship were required to participate in the clinical performance encounter.

### SP Preparation

Two SP educators collaborated in an initial session with two experienced SPs to provide feedback to clerkship leadership on the encounter prior to utilizing this teaching tool with students. Once the SP encounter had been finalized, it was provided to the SPs for home study prior to a 2-hour training session on site (Appendix A). We trained five SPs for this scenario. SPs with a history of pairing well together and scoring similarly were chosen to help ensure standardization. The clerkship director and two SP educators partnered to lead the on-site training and conduct role-plays with the SPs. This discussion and role-play opportunity allowed the SPs to further fine-tune and standardize the portrayals. Two SPs were present during each session, allowing the eight students to rotate between the dyspareunia case and their second obstetrics and gynecology case during the two 30-minute sessions. On the day of each session, the SPs reported 30 minutes prior to the start of the event for further discussion and to address any outstanding questions. At the end of each session, the SPs debriefed with the coordinating SP educator to document any unexpected questions that could benefit from additional standardization. Videos of actual sessions with students were subsequently used to train new SPs and were interrated to again ensure standardization. We set up the simulation center as an outpatient clinic. No props or additional materials were needed for this encounter.

### Patient Encounter

Upon arrival, students received information regarding the case and the tasks they needed to complete during their encounter as a door note prior to entering the encounter (Appendix B). Students were told that their SP, June Bellavue, was presenting as a new patient to their outpatient clinic because she had “pain with intercourse.” Students were given 15 minutes with their SP. As students have other teaching sessions to learn and receive feedback on their pelvic exam skills, the SP encounter focused solely on history taking and counseling of the patient based on the history obtained and the limited physical exam information provided (vital signs and results of a pelvic exam from a prior provider). Our simulation center had the capacity to videotape each student during the encounter. Our simulation center gave timed cues for the students for a 5-minute warning and at the end of the encounter. Two simulation center staff members were available during the encounters to troubleshoot any questions that came up from a student or SP. Upon finishing their encounter, students had 10 minutes to complete a postencounter note (Appendix C).

While the student completed the postencounter note, the SP filled out a set evaluation of the student (Appendix D). This evaluation was created by the Duke University School of Medicine and was consistent across all SP encounters in the simulation center. As our SPs had specific training to provide feedback on student interpersonal skills, the long-form evaluation included different aspects of the encounter to provide specific comments to the students on where they excelled and potential areas for improvement in their interpersonal skills. We also asked students to evaluate themselves on their interaction with the SPs and ability to put their SP at ease during the encounter (Appendix E), mirroring the first aspect of the SP evaluation. Students reentered the room after finishing their postencounter note to discuss the SP's evaluation for 5 minutes, completing the full 30 minutes required for the encounter. All videotapes from the individual SP encounters and written documentation including the postencounter note and evaluation were immediately uploaded to LearningSpace, a secure web-based simulation center management tool to compile, score, and review both notes and videos related to the SP encounter (CAE Healthcare, 2019). While this tool allowed our simulation center staff to manage student data from the encounter and obtain feedback from both students and faculty regarding student performance, the SP encounter can be implemented without use of the software program.

After the encounter was complete, students were given web-based access to their videotaped encounter. We instructed the students to watch their encounter to determine which elements of the sexual history they had elicited (Appendix F). Students evaluated themselves again on their interaction with the SPs after watching their videotaped encounter using the same evaluation tool used immediately after their SP encounter (Appendix E). For programs that do not have videotape capacity, Appendix E can be omitted from the exercise.

### Summary Didactic Session

In response to feedback from medical students requesting an opportunity to debrief the SP encounter and a more formalized curriculum on the differential diagnosis for a patient with dyspareunia, we developed a corresponding summary didactic session. This session provided a patient presentation similar to that of their SP, followed by a differential diagnosis and overview of different causes of dyspareunia with practice multiple-choice test questions (Appendix G). This didactic session was created by the clerkship director and a resident with interest in medical education. Students were provided with the summary didactic session handout and reviewed the content with a faculty mentor during dedicated didactic time the week immediately after the student had completed the SP encounter. This session was conducted in an interactive fashion, with students thinking through the case presentation and two guiding questions prior to discussing the differential diagnosis and treatment modalities for dyspareunia. Students completed comprehension questions at the end of the session and subsequently reviewed their answers with the didactic group to evaluate their understanding of the material presented. This summary didactic session was held with the students' didactic group for the clerkship, which was approximately 10 students in size. Faculty mentors had previous mentorship and education on how to conduct case-based learning exercises with the didactic groups. We did not give additional training for this specific case, although it was designed in the same framework as all the didactic cases on which faculty had been previously trained. Of note, all faculty were OB/GYN providers with experience in management of this topic. Faculty mentors were advised to reach out to the clerkship director for any issues that came up during the summary didactic session. Students were not graded on their performance in this session or their answers to the comprehension questions following the session, as the session was created solely for the purpose of providing additional learning on dyspareunia following the SP encounter and assessment. However, students were expected to participate in this planned didactic session as part of their professionalism grade, which accounted for 5 of the 100 points towards the overall clerkship score.

### Learner Assessment

The SP encounter was created to be a formative experience halfway through the clerkship. Students received real-time verbal and written feedback from their SP regarding their history and physical skills, including specific feedback on the SP comfort while the student was discussing sexual health. Students received feedback on their postencounter note (Appendix C) by receiving half a point for each aspect of the history of present illness and physical exam that they elicited, followed by 1 point each for obtaining a possible correct differential diagnosis, providing supporting evidence for their differential diagnosis, and offering a possible management plan. The focus of this exercise was to provide feedback predominantly on the history of present illness; thus, more points were possible in this section of the rubric. While other topics that could affect dyspareunia, such as intimate partner violence and mood disorders, were not directly attributed points in the rubric, students obtained points for asking about these subjects through the social history, medications, OB history, and GYN history aspects of the postencounter note. Students were also graded on their ability to elicit specific details of the SP's sexual history based on their video review of their SP encounter (Appendix F). Students received 1 point for each aspect of the sexual history they elicited, up to 13 total points, based on previous existing resources regarding information that should be obtained a sexual history.^16–19^ The postencounter note, grading rubric, and sexual history questions were created by the clerkship director, who had formal training in undergraduate medical education, and were reviewed by a working group including two physicians in obstetrics and gynecology with backgrounds in medical education, one physician in obstetrics and gynecology with a professional interest in dyspareunia, and one physician assistant practicing general gynecology.

A team of residents and faculty from the obstetrics and gynecology department with an interest in education volunteered to help review and score the postencounter learner note (Appendix C). Residents and faculty involved in scoring these encounters received training in how to navigate the web-based note system and specific grading criteria based on aspects of the history and physical that the student reported and the student's reported differential diagnosis and treatment options. Residents and faculty were able to bring any questions or concerns regarding grading to the clerkship director, who created the SP encounter and grading rubric. We determined numerical grades for students as a point value summation of scores for Appendices C, D, and F (hereafter referred to as the SP encounter raw score); though these assessments did not have any influence on their final grades for the clerkship. Students also received individual scores for each part of the SP encounter.

### Evaluation

Evaluation of the SP encounter was conducted through analysis of student and SP data collected on the web-based management tool. Data from postencounter learner notes, postencounter SP evaluations, postencounter learner observation, and postencounter learner evaluations were analyzed. Summary statistics of continuous variables (mean, standard deviation) and categorical variables (percentage) were calculated as reported below. Deidentified characteristics of students were obtained from the clerkship coordinators, including timing of the student's obstetrics and gynecology clerkship within the overall clerkship year, clerkship subrotation prior to the SP encounter (obstetrics, gynecology, or outpatient), and subsequent clerkship grade. Summary statistics of the SP encounter were stratified by these student characteristics.

Multiple linear regression models were used to determine the effects of any of the student characteristics on the SP encounter scores, as well as the effects of the SP encounter scores on the shelf score and overall clerkship score. Analysis of variance (ANOVA) models were used to compare average SP encounter scores based on rotation letter grade. Data analysis was performed with Stata 15.1 (StataCorp, 2017) at a significance level of .05, two-tailed.

Qualitative feedback on the SP encounter was collected by the clerkship leadership via student feedback during the mid-rotation feedback and end-of-rotation evaluation. Clerkship leadership recorded quotes from students during mid-rotation feedback; these were then compiled to determine trends. Furthermore, students rated how well the SP encounter enhanced their learning and identified areas of weakness in their knowledge on their end-of-clerkship evaluation. These results were compared to other educational experiences during the clerkship. The end-of-rotation evaluation was administered by the Duke University School of Medicine, and results were compiled in aggregate and released every 6 months to clerkship leadership. Given the lag in clerkship feedback to allow for deidentification, end-of-clerkship data are presented for student participants from the first half of the academic year only.

## Results

### Participant Characteristics

We implemented this SP case with 101 second-year medical students at the Duke University School of Medicine. An additional 16 students completing the obstetrics and gynecology clerkship as part of a longitudinal integrated program were not included in the analysis. Students' overall clerkship score was based on their clinical performance, professionalism, and final examination (shelf) score. Information about the learners can be found in Table 1.

**Table 1.**
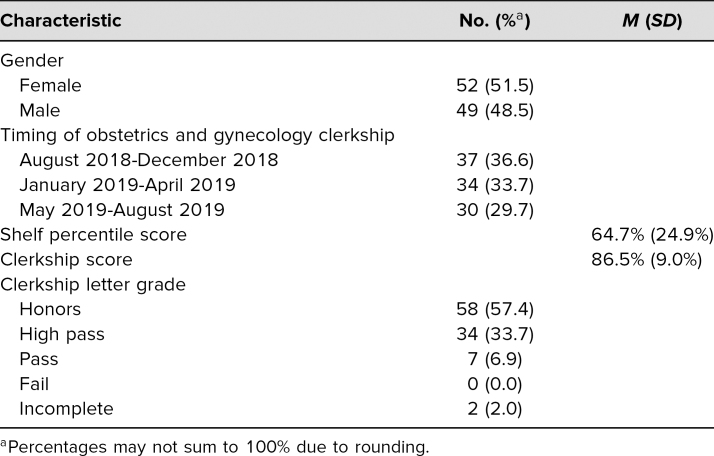
Student Characteristics (*N* = 101)

### Overall SP Encounter Results

The average SP encounter raw score was 60.2 out of 100 (*SD* = 11.2). Students scored highest on their postencounter SP evaluation (77.3, *SD* = 13.0) and lowest on their postencounter learner note and video review of their sexual history taking (31.9, *SD* = 11.2, and 30.4, *SD* = 13.4, respectively). Notably, the SP encounter raw score was not affected by the clerkship subrotation preceding the SP encounter, even when adjusting for rotation timing (unadjusted ß = 0.7, 95% CI = −1.9 to 3.2, *p* = .6; adjusted ß = 0.9, 95% CI = −1.2 to 3.0, *p* = .4).

### Differential Diagnosis for Dyspareunia

On the learner postencounter notes, students documented an average of 4.8 of 12 key aspects of the patient's sexual history (*SD* = 1.5). As students were determining a differential diagnosis, 67 (66%) of students identified one of the most likely diagnoses for the SP's symptoms (inhibited arousal, vaginismus, vulvodynia, medication side effect, vulvovaginitis, hypothyroidism), although almost all (62, 92%) of those provided supporting evidence for their diagnosis (Table 2). Similarly, only 49 (48%) students provided an appropriate management plan based on their differential.

**Table 2.**
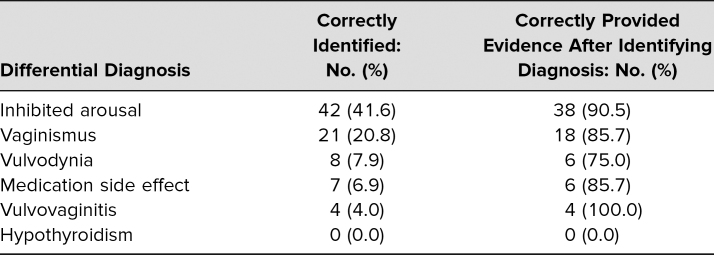
Dyspareunia Differential Diagnosis on Postencounter Learner Note (*N* = 101)

### Sexual History Taking

Both SPs and students evaluated the environment the student created to discuss the patient's sexual complaint, rated on a 5-point scale (1 = *poor,* 2 = *fair,* 3 = *adequate,* 4 = *very good,* 5 = *excellent*). Students were rated as above average by themselves and their SPs across all aspects of creating a safe environment to discuss sexual complaints. Furthermore, SPs rated students as very good or excellent more often than the students themselves did (Table 3).

**Table 3.**
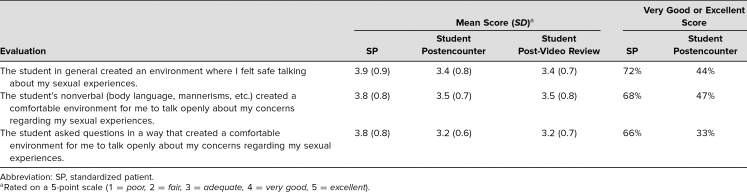
Student History Taking Skills Scored by SP and Self-Evaluations (*N* = 101)

Of the 13 aspects of the sexual history we provided to the SP, students elicited on average 3.9 of those aspects after rewatching their encounter on videotape (*SD* = 1.8). The most common aspects of the sexual history that were elicited were use of lubrication (*N* = 82, 81%) and age of sexual debut (*N* = 78, 77%). After that, specific questions fell off markedly (the next most common response was amount of time with current partner, *N* = 42, 42%). Only 37% of students asked about gender of sexual partners, 35% about number of lifetime partners, and 36% about history of physical or sexual abuse. Fifteen percent of students asked about either oral intercourse or anal intercourse, and only 7% about ability to orgasm.

### Correlation of SP Session With Clerkship Performance

There was no relationship between the total SP encounter score and the shelf percentile score, even when controlling for pre-SP encounter rotation and rotation timing (unadjusted ß = 0.05, 95% CI = −0.4 to 0.5, *p* = .8; adjusted ß = 0.07, 95% CI = −0.5 to 0.6, *p* = .8). Total SP encounter score was initially associated with the final rotation score, but this relationship was not statistically significant after controlling for pre-SP encounter subrotation and rotation timing (unadjusted ß = 0.2, 95% CI = 0.1–0.4, *p* = .009; adjusted ß = 0.1, 95% CI = −0.1 to 0.3, *p* = .2). Similarly, an ANOVA of SP encounter score by the final clerkship letter grade was not statistically significant (*p* = .1).

### Participant Feedback

Students were allowed to comment on the SP encounter during their midpoint clerkship feedback session and on their end-of-clerkship evaluation. Of note, this SP encounter was paired with another SP encounter on abnormal uterine bleeding. Feedback was directed towards the SP experience as a whole and was not separated into the two separate SP encounters.

Students overall reported positive experiences with the SP encounter and appreciated that they had the opportunity to receive formative feedback in a low-stress environment. Some students noted that they felt unprepared prior to the SP encounter but that it helped them to identify gaps in their learning moving forward. Students noted that they had few other opportunities to practice taking a sexual history, and they appreciated the time to counsel their patients.

At the end of the clerkship, students indicated that the SP encounter as a whole was helpful in enhancing learning and identifying areas of weakness in knowledge. Most students indicated that the SP encounter helped “adequately well” or better in both of these areas (62% reported enhanced learning, 72% reported identified knowledge gaps). Additionally, the SP experience appeared similar to other experiences in the clerkship in terms of its overall educational value, with 30% of students rating the SP experience as above average or excellent as a learning experience. Two students mentioned the SP encounter as the most valuable aspect of the clerkship in a free-response section in the end-of-clerkship evaluation.

## Discussion

We present the first year of data for the development and utilization of an SP case and corresponding summary didactic session on dyspareunia at a single academic medical center. This SP case provided medical students with the opportunity to ask hard, sometimes uncomfortable, and detailed questions about a woman's sexual past. We found that overall, our students had strong interpersonal skills and were able to effectively ask questions about a sexual history but had little prior clinical knowledge of the types of questions to elicit a sexual history as well as possible differential diagnosis or management options for patients with sexual complaints. Scores on the SP encounter were not associated with shelf exam score, although there was a correlation between the SP encounter raw score and the final clerkship grade. This was not statistically significant after controlling for other variables.

Our findings on the lack of comfort with sexual history taking and causes of dyspareunia are consistent with other studies on sexual history taking among medical students and point to the need for this case to ensure students have exposure to this topic during their medical training.^4^ However, students indicated that the feedback they received from this session was appreciated and helpful in advancing their skills. Indeed, other SP encounters for sensitive subjects, such as pelvic exam skills, have enhanced student experience through directed immediate feedback and understanding of the doctor-patient relationship in a low-pressure environment.^20^ Additionally, similar SP cases are being used across other medical disciplines for topics not well covered by the standard curriculum, such as LGBTQI care.^21^

Furthermore, we illustrate a possible correlation between SP encounter score and final clerkship grade, although more data are needed to understand the influence of this SP encounter on student performance overall. While the SP encounter offers formative feedback compared to shelf scores and overall clerkship scores, which are summative feedback for students in the OB/GYN clerkship, clinical knowledge associated with this SP encounter may be a good marker for self-directed learning among the OB/GYN clerkship students. Students are expected to engage in their own learning outside of clinical time based on the APGO curricular objectives, and clinical knowledge related to dyspareunia may be associated with clinical knowledge of other topics tested in the shelf exam or throughout the clerkship. Moreover, because it features an orphan topic that brings together many other key aspects of OB/GYN—understanding pelvic anatomy, performing an appropriate OB/GYN review of systems, and the ability to ask sensitive questions—performing well on this SP encounter may be a marker for students who perform well in the clerkship overall. Finally, we used SP comments to identify any red flags in patient interactions to help provide struggling students with additional support through the rest of the clerkship.

While our current evaluation tools did not indicate a correlation between the SP encounter and more traditional summative evaluations in the OB/GYN curriculum, SP encounters and simulation curricula have shown efficacy over the years in improving clinical knowledge among trainees.^8,22^ Due to logistics of the current SP and didactic sessions, we were unable to assess whether this SP encounter impacted individual patient encounters with students after completing the case. Additionally, feedback from students regarding the SP encounter included both this encounter as well as an abnormal uterine bleeding encounter conducted simultaneously, thus making it more challenging to determine the individual value of the dyspareunia SP encounter compared with other teaching modalities in the clerkship. Alternative methods for evaluation of students, including longer-term assessment of student retention or use of these skills in later clerkships, may be helpful in determining the value of this SP encounter for student medical training.

Even with lack of specific data relating the SP encounter to shelf or clerkship scores, verbal feedback from students indicated that this addition to their curriculum did enhance their clinical encounters. This student feedback highlighted that participants used their experience with this SP encounter to identify their own personal gaps in knowledge, prompting a request for more formal teaching on the subject, which was subsequently filled through the summary didactic session. This catalytic effect of the SP encounter to drive future learning forward speaks to its capacity as a good assessment tool and argues for its continued use in the clerkship.^23^

In developing and implementing this case, we felt that we effectively bridged a gap within our clerkship. Through the course of implementation, we realized the need for a debriefing opportunity after the case. Initially, we had thought that review of a videotaped encounter as well as formal feedback on students' notes would be adequate for this content. However, after the first rotation, nearly all students requested more formal didactic teaching on the topic. Therefore, we developed the above summary didactic session. After this, students expressed a better understanding of both the clinical material and the history taking skill. We also have since realized that the didactic session could easily be used alone (without the SP case) if resources were limited.

There are limitations to our study. To date, this SP case has been used only at a single academic medical center with a well-organized clinical skills lab. This may limit generalizability, as the case would be more challenging at centers without this resource. Furthermore, as previously detailed, our clinical skills lab uses a secure web-based management tool to organize student notes and videos related to the SP encounter, as well as to compile feedback from faculty, SPs, and student self-reflection. In the absence of this technology, feedback could be given via direct observation, and notes could be written in any word-processing program and sent to evaluators. We have noted in the Methods section other modifications to the activity that can also be made depending on resources of individual institutions. Finally, our assessment of students' comfort with sexual history taking and dyspareunia was at only one time point and did not control for prior experiences students may have had in this topic area. Many students indicated that they focused more on this area of the curriculum after their SP encounter, so further studies could assess students' knowledge and comfort both at the time of the SP encounter and at the end of the clerkship through a pre-post survey design or longer-term follow-up of these students.

In conclusion, this SP case was effective in exposing students to patients with sexual pain and allowing them to take a sexual history, a prior gap in our clerkship curriculum. This SP case allowed students direct clinical experience of employing communication skills to discuss an often-uncomfortable topic, while also providing didactic teaching through the summary didactic session. Ensuring all medical providers, not just gynecologists, feel comfortable with this topic is critical for the sexual health of women. The case was easily employed with all medical students on their obstetrics and gynecology clerkship and received good overall feedback as to its utility. As we continue to refine this SP case, we will continue to gather data on student performance as well as student feedback. Given the effectiveness of the case in covering a topic that is important but infrequently encountered clinically by our students, we are considering developing SP cases on other such topics, specifically, menopause, to address gaps in our curriculum.

## Appendices

Preencounter SP Information.docxPreencounter Learner Information.docxPostencounter Learner Note.docxPostencounter SP Evaluation.docxPostencounter Learner Evaluation.docxPostencounter Learner Observation.docxSummary Didactic Session.docx
All appendices are peer reviewed as integral parts of the Original Publication.
