# Supplementary material for: Understanding Sexual Complaints and History Taking: A Standardized Patient Case on Dyspareunia for Obstetrics and Gynecology Clerkship Students
Source: MedEdPORTAL. 2020 Oct 29;16:11001. doi: 10.15766/mep_2374-8265.11001 (PMC7597941; doi:10.15766/mep_2374-8265.11001)
Supplement: Supplementary file 1 — Preencounter SP Information.docxPreencounter Learner Information.docxPostencounter Learner Note.docxPostencounter SP Evaluation.docxPostencounter Learner Evaluation.docxPostencounter Learner Observation.docxSummary Didactic Session.docx [file mep_2374-8265.11001-s001.zip › A. Preencounter SP Information.docx]

Appendix A: Preencounter SP Information

Primary Case Author: Jill Hagey, MD MPH

Name of Case: Dyspareunia

Name of educational and or assessment activity: Clinical Performance Examination (CPX)

Patient Name: June Bellavue

Chief Complaint: “It hurts when we have sex”

Most likely Diagnosis and Differential with rationale from history and/or physical exam:

1. Inhibited arousal leading to inadequate lubrication
2. Vaginismus
3. Vulvodynia
4. Vulvovaginitis
5. Medication side effect
6. Hypothyroidism

Challenge question:

Domains: Check all that apply

⌧ Professionalism

⌧ Communication and Interpersonal skills

⌧ Medical History

🞎 Physical exam

⌧ Shared Decision Making

⌧ Patient Education

⌧ Clinical Reasoning

⌧ Documentation

🞎 Handoff

🞎 Presentation

🞎 Other:

Type and level of learner: First year clerkship medical students in obstetrics and gynecology rotation

Case Objectives: please list specific objectives for each of the domains you have checked above:

1. List three to five diagnoses that may lead to dyspareunia and explain the defining features of each diagnosis.

2. Use verbal and non-verbal cues to create a comfortable environment for a standardized patient to share information about her sexual history and sexual concerns.

3. Engage a standardized patient in discussion of treatment options for dyspareunia, including responding to specific concerns voiced by the patient.

| SETTING: outpatient, in patient, ED, home, nursing home, rehab, group etc. | Outpatient |
| --- | --- |
| PATIENT PROFILE: Information about the “patient” that helps select an SP and helps the learner get an understanding of them as a person. SP will know more information about the patient than learner will ever ask but allows SP to portray a fully developed patient personality. If none of the items below are particulars for the case please write “all may be used.” | |
| Age range | 18-45 |
| Religious/spiritual background | Christian |
| Sex (e.g., male, female, intersex, transwoman, transman) | Female |
| Sexual Orientation (e.g., heterosexual, lesbian, gay, bisexual, pansexual, queer, asexual) | All may be used |
| Gender expression (e.g., man, woman, gender queer) | All may be used |
| Race/ethnicity: | All may be used |
| Physical description (e.g., BMI, height range) | All may be used |
| Physical limitations | All may be used |
| Patient appearance (e.g., disheveled, hospital gown, business casual, casual) | Casual |
| Moulage + location (e.g., none, bruises, scars, body piercing, tattoos) | None |
| Affect (e.g., pleasant, cooperative) | Nervous, cooperative |
| Family group (e.g., who is family, who they live with) | Family: Daughter, age 3; Boyfriend (father of daughter) |
| Education | College-level education |
| Level of health literacy | Moderate |
| Employment, if any - present and past, noting any current stresses | Works at University of North Carolina as a Health Unit Coordinator on the Neurology floor |
| Home/homeless - type of dwelling, number of stories, owned or rented | Housed, rents a 2-bedroom home with her boyfriend in Durham |
| Financial situation- any current stresses | “Middle class,” has steady income from her job |
| Insurance Status (e.g., un/under/insured, public/private, HMO/PPO) | Insured |
| Habits (i.e., diet, exercise, caffeine, smoking, alcohol, drugs) | Has never smoked or used tobacco products. Has alcohol at special occasions, less than once a month. Tries to eat healthy, gave up soda last year and fast food for Lent last year. Walks 3-4 times a week (20-30 minutes), considering trying yoga. |
| Activities (i.e., hobbies, sports, clubs, friends) | Enjoys cooking with boyfriend, also teaches Sunday school to 3^rd^ graders |
| Typical day - what is the usual daily routine |  |

| CASE INFORMATION | |
| --- | --- |
| Chief Concern: What the patient will say when greeted by the student. The patient’s primary reason for seeking medical care often stated in his/own words. | “It hurts when we have sex” |
| Additional Concerns: Other, if any, concerns the patient has today (i.e., symptoms, requests, expectations, etc.) that will become part of set agenda. | A little nervous to talk about “sex life”, but also optimistic that provider might be able to help/make sex better. |
|  | |
| THE PATIENT STORY: The SP will be asked to tell their symptom story and the personal and emotion impact for each of their concerns. You will want to write this is the patient voice. The symptom story should be able to answer this question: “Tell me more about [chief concern/additional concern], starting at the beginning and bringing me up to now.”  The personal context should be able to answer questions concerning the broader personal/psychosocial context of symptoms, especially the patient beliefs/attributions.  The emotional context should be able to ask how are you doing with this, how does this make you feel, how has this affected you emotionally? IMPACT: How has this affected your life? How has this been for your family? | I’ve always had pain when having sex. I noticed it since I started having sex when I was 17 and thought that it would get better, but the pain has continued since then and it is affecting my relationship with my boyfriend. The pain feels like I’m being rubbed raw, almost a burning sensation. It is worse when we start having sex, but it continues throughout the experience and I’m even sore for a few hours afterwards. It was supposed to get less painful, but it never really has. |
| HISTORY OF PRESENT ILLNESS: Although some of the HPI will be given in the patient’s symptom story, the learners will expand the story during the direct question section. Below describe the detailed history, usually about the chief concern, which the student must develop in order to make a useful assessment of the problem: | |
|  | |
| Onset (when; gradual or sudden) | Only during intercourse, gradual onset |
| Setting (what was going on or where was patient when symptoms first noticed?) | Happens with penetration, or after sex has been going on a little while |
| Duration (how long) | During intercourse. Sometimes, I’m even sore for a few hours after we have sex. |
| Time relationships (frequency, constant or intermittent) | I’ve always had pain with sex. They said it would get better after the first time, but it never really did. |
| Location | Vagina. It hurts “on the outside part” and a little “in the inside.” |
| Radiation | None |
| Quality | Raw, burning irritation |
| Amount | Hard to quantify on a 1-10 scale. Never had to stop because of the pain. |
| Aggravated by what | With intercourse only, it’s not really worse any specific time of the month. No different after having a baby. |
| Relieved by what | KY jelly helps at the beginning, but it still is sore at the end. If we reapply, that helps, but it’s hard to “kill the moment” and my boyfriend feels bad when we use it because he feels like “he isn’t doing his job.” Sometimes, it hurts a little less when I’m “more into it” though. |
| Associated with what | No malodorous discharge, no pain or burning with urination, no vaginal itching. No bleeding with intercourse. |
| Attitude (what does the patient think is the problem, and how does he/she feel about it) | It really effects for my quality of life. My boyfriend feels bad because it really hurts. |
| Overall course |  |
| REVIEW OF SYSTEMS: Significant positives and negatives | |
| General | Negative |
| Cardiac | Negative |
| Respiratory | Negative |
| Neurologic | Negative |
| Digestive | Negative |
| Genital | See HPI |
| Skin | Negative |
| Breast | Negative |
| Pysch | Negative |
|  | |
| Past medical history |  |
| Medication allergies (Name and reaction) | None |
| Environmental allergies (Name and reaction) | None |
| Illnesses | Mild depression |
| Vaccinations | Up to date except did not get HPV vaccine |
| Surgeries | Dilation and curettage for incomplete abortion |
| Accidents/ injuries/ trauma | None |
| Hospitalization | Delivery of baby |
|  | |
| Inclusive sexual and reproductive history | |
| Sexual practices  Sexual partners  Protection: Use of safer sex practices  Use of birth control if appropriate  Risk of intimate partner violence | Oral sex and vaginal sex, denies anal sex, occasionally uses vibrator for masturbation  5 lifetime male partners, has had current partner for 4 years; sexual debut at age 17  Previously used condoms; switched away from condoms due to fear of latex allergy; history of trichomonas with first partner as a teen but no STI since  Uses condoms occasionally, on oral contraceptive pills  Feels safe with current partner, no history of physical, emotional or sexual abuse |
| Ob/GYN HISTORY | Age of onset of menses: 12  Age of menopause: N/A  Number of pregnancies: 2  Number of live births: 1  Number of miscarriages: 1 (incomplete miscarriage resulting in dilation and curettage)  Number of abortions: 0 |
| Medications | Prescription/dose/reason: Zoloft 25mg daily (mild depression), Loestrin daily (birth control)  Over the counter/dose/reason: Motrin 600mg (as needed for pain)  Herbs/supplements/dose/reason: Multivitamin  Other: |
| Immunizations | ⌧ Tetanus  ⌧ Flu  ⌧ Hepatitis  🞎 Pneumovax  🞎 HPV  🞎 Other |
| Tobacco products:  🞎 Cigarettes  🞎 Cigar  🞎 Pipe  🞎 Chew  🞎 E-cigarettes | ⌧ Never  🞎 Past- year started/year quit  🞎 Current   - - Quantity   - # of years |
| Alcohol  ⌧ Beer  ⌧ Wine  🞎 Liquor  🞎 Other | 🞎 Never  🞎 Past- year started/year quit  ⌧ Current   - - Quantity: 1 glass/month   - # of years: 5 |
| Drugs  🞎 Weed  🞎 Cocaine  🞎 Heroin  🞎 Meth  🞎 Other  🞎 IV  🞎 Inhalants  🞎 Other | ⌧ Never  🞎 Past- year started/year quit  🞎 Current   - - Quantity   - # of years |
| Diet (describe) | Tries to eat healthy, gave up soda last year and fast food for Lent last year. |
| Exercise (describe) | Walks 3-4 times a week (20-30 minutes), considering trying yoga. |
| List any other important social history or information important to this case | N/A |
| Family history | See below |
| Mother, Father, Siblings, Grandparents, and other significant findings. | Father: Age 65, Hypertension  Mother: Age 62, Breast cancer (age 61, in remission)  Sister: Age 35, Type 2 Diabetes  Daughter: Age 3, Healthy |
|  |  |
| Physical Exam- List exam maneuvers expected for this case and any abnormal findings that SP will simulate. (tenderness, hyper-hypo reflex, rebound, weakness etc. )  General appearance: well appearing, no apparent distress, slightly nervous on answering questions | |
| PHYSICAL EXAM FINDINGS |  |
| 1. Written in layman’s terms | No abnormal findings; physical exam not performed |
| 1. General appearance- affect, appearance, position of patient at opening (i.e. sitting, laying down, holding abdomen etc.) | Sitting on a chair, engaged but slightly nervous demeanor |
| 1. Vital signs | Normal |
| 1. Specific findings and affect | None |
| 1. Response to certain physical movements | None |
|  |  |
| DIAGNOSIS AND DIFFERENTIAL |  |
| Diagnosis with support from positive and negative history and PE findings |  |
| Differential with support from positive and negative history and PE findings | 1. Inhibited arousal leading to inadequate lubrication 2. Vaginismus 3. Vulvodynia 4. Vulvovaginitis 5. Medication side effect 6. Hypothyroidism |
|  |  |
| MANAGEMENT OR DIAGNOSITIC PLAN | 1. Offer/refer to pelvic physical therapy 2. Validation of pain/discussion of foreplay and other arousal techniques 3. Review lubricant options/how and when to use 4. Offer/refer to sex therapist 5. Discussion of changing SSRI to other antidepressant with different side effect profile 6. Discussion of changing OCPs to other contraception with different side effect profile |
|  |  |
| PROFESSIONALISM ISSUES OR CHALLENGES: | None |
