# Supplementary material for: Understanding Sexual Complaints and History Taking: A Standardized Patient Case on Dyspareunia for Obstetrics and Gynecology Clerkship Students
Source: MedEdPORTAL. 2020 Oct 29;16:11001. doi: 10.15766/mep_2374-8265.11001 (PMC7597941; doi:10.15766/mep_2374-8265.11001)
Supplement: Supplementary file 1 — Preencounter SP Information.docxPreencounter Learner Information.docxPostencounter Learner Note.docxPostencounter SP Evaluation.docxPostencounter Learner Evaluation.docxPostencounter Learner Observation.docxSummary Didactic Session.docx [file mep_2374-8265.11001-s001.zip › B. Preencounter Learner Information.docx]

Standardized Patient CPX Student Encounter: Dyspareunia

**Preencounter Learner Information**

**Patient Name:** June Bellevue

**Setting:** Outpatient clinic

**Patient Information:**

Ms. Bellavue is a reproductive aged female who presents as a new patient to your clinic for “pain with intercourse.” She is referred from her family doctor who she saw last week for a well woman exam, at which time, her speculum and bimanual exams were normal.

**Vitals:**

Blood Pressure: 126/72

Pulse: 76

Temperature: 98.3 F

Respiration: 18

**Your Tasks:**

Counsel patient on possible etiologies of her pain. In the **15 minutes** with the patient:

- Obtain a problem-focused history that includes an OBGYN and sexual history
- Counsel the patient on possible etiologies of her pain, including potential diagnostic tests or management strategies you may use to help her symptoms based on these possible etiologies

**DO NOT PERFORM A PHYSICAL EXAM**

In the remaining **10 minutes**:

- Write a note that includes your medical decision-making, including a prioritized differential and a proposed workup/management plan
