# Supplementary material for: Understanding Sexual Complaints and History Taking: A Standardized Patient Case on Dyspareunia for Obstetrics and Gynecology Clerkship Students
Source: MedEdPORTAL. 2020 Oct 29;16:11001. doi: 10.15766/mep_2374-8265.11001 (PMC7597941; doi:10.15766/mep_2374-8265.11001)
Supplement: Supplementary file 1 — Preencounter SP Information.docxPreencounter Learner Information.docxPostencounter Learner Note.docxPostencounter SP Evaluation.docxPostencounter Learner Evaluation.docxPostencounter Learner Observation.docxSummary Didactic Session.docx [file mep_2374-8265.11001-s001.zip › C. Postencounter Learner Note.docx]

Standardized Patient CPX Student Scoring Criteria: Dyspareunia

**Postencounter Learner Note**

HISTORY: Describe the history you just obtained from this patient. Include only information (pertinent positives and negatives) relevant to this patient's problem(s).

**Scoring (0.5 points for each)**

1. Chief Complaint
   1. Location: vulva/introitus mostly, some deeper vaginal pain
   2. Quality: raw, burning, irritating
   3. Severity: moderate, causes distress with sex life
   4. Onset: since sexual debut
   5. Duration: occurs with penetration and sometimes after sex for a few hours as well
   6. Has had with all partners, no different with current partner
   7. No changes after having baby, no cyclic differences related to menstrual cycle
   8. Associated symptoms: no bleeding with intercourse, no hot flashes, no vaginal discharge
2. Medications
3. OB History
4. GYN/Sexual History
5. Social History

PHYSICAL EXAMINATION: Describe any positive and negative findings relevant to this patient's problem(s). Be careful to include only those parts of examination you performed in this encounter.

**Scoring (0.5 points for each)**

1. Vitals/general appearance
2. Abdominal Exam

DATA INTERPRETATION: Based on what you have learned from the history and the physical examination, list up to 3 diagnoses that might explain this patient's complaint(s). List your diagnoses from most to least likely. For some cases, fewer than 3 diagnoses will be appropriate. Then, enter the positive or negative findings from the history and the physical examination (if present) that support each diagnosis.

**Scoring (1 point for diagnosis, 1 point for supporting evidence)**

1. Inhibited arousal leading to inadequate lubrication
2. Vaginismus
3. Vulvodynia
4. Vulvovaginitis
5. Medication side effect
6. Hypothyroidism

DIAGNOSTIC STUDIES/MANAGEMENT PLAN: Based on your differential diagnosis, list initial diagnostic studies (if any) you would order for each listed diagnosis (e.g. restricted physical exam maneuvers, laboratory tests, imaging, ECG, etc.). You may also list any management plan you would offer to the patient based on your differential.

**Scoring (1 point for each)**

1. Offer/refer to pelvic physical therapy
2. Validation of pain/discussion of foreplay and other arousal techniques
3. Review lubricant options/how and when to use
4. Offer/refer to sex therapist
5. Discussion of changing SSRI to other antidepressant with different side effect profile
6. Discussion of changing OCPs to other contraception with different side effect profile
