# Supplementary material for: Understanding Sexual Complaints and History Taking: A Standardized Patient Case on Dyspareunia for Obstetrics and Gynecology Clerkship Students
Source: MedEdPORTAL. 2020 Oct 29;16:11001. doi: 10.15766/mep_2374-8265.11001 (PMC7597941; doi:10.15766/mep_2374-8265.11001)
Supplement: Supplementary file 1 — Preencounter SP Information.docxPreencounter Learner Information.docxPostencounter Learner Note.docxPostencounter SP Evaluation.docxPostencounter Learner Evaluation.docxPostencounter Learner Observation.docxSummary Didactic Session.docx [file mep_2374-8265.11001-s001.zip › D. Postencounter SP Evaluation.docx]

Standardized Patient CPX Student Scoring Criteria: Dyspareunia

**Postencounter Standardized Patient Evaluation**

|  | Poor | Fair | Adequate | Very Good | Excellent |
| --- | --- | --- | --- | --- | --- |
| 1. The student in general created an environment where I felt safe talking about my sexual experiences | ( ) | ( ) | ( ) | ( ) | ( ) |
| 2.The student’s non-verbal (body language, mannerisms etc) created a comfortable environment for me to talk openly about my concerns regarding my sexual experiences | ( ) | ( ) | ( ) | ( ) | ( ) |
| 3.The student asked questions in a way that created a comfortable environment for me to talk openly about my concerns regarding my sexual experiences | ( ) | ( ) | ( ) | ( ) | ( ) |

**Interpersonal**

| Interpersonal skills reflect a student’s ability to provide an effective exchange of information and develop a therapeutic relationship with their patients. They are a combination of communication skills (questioning and information-sharing) and relational skills (empathy and respect). The components of interpersonal skills being evaluated in this exam are: | | | | | |
| --- | --- | --- | --- | --- | --- |
| 4.  Introduction | ( )  Poor: *Does not introduce self *Does not identify you by name *Disinterested greeting | ( )  Fair | ( )  Adequate: *Introduces self *Identifies you by name *Appropriate greeting | ( )  Very Good | ( )  Excellent: *Introduces self by name and position *Identifies you by name *Warm and engaging greeting |
| 5.  Questioning Skills | ( )  Poor: *No use of open-ended questions *Multiple interruptions *Scattered and disjointed line of questioning | ( )  Fair | ( )  Adequate: *Some use of open-ended questions *Few interruptions *Basic flow to line of questioning | ( )  Very Good | ( )  Excellent: *Effective use of multiple open-ended questions *Zero to minimum interruptions *Smooth transitions and natural flow |
| 6.  Elicit Patient Perspective | ( )  Poor: *Uninterested in your explanatory model *Unconcerned with how illness may affect well-being *Resistant to incorporating your viewpoints into decision making | ( )  Fair | ( )  Adequate: *Acknowledges your explanatory model *Receptive to your concerns over impact of illness on well-being *Incorporates your input into decision making | ( )  Very Good | ( )  Excellent: *Proactively assesses your explanatory model *Explores your concerns over impact of illness on well-being *Proactively solicits your viewpoint in decision making |
| 7.  Verbal Communication | ( )  Poor: *Uses medical jargon excessively *Thoughts consistently disorganized *Tone of speech conveys indifference or detachment | ( )  Fair | ( )  Adequate: *Limited use of medical jargon *Most thoughts are well organized *Comfortable tone of speech | ( )  Very Good | ( )  Excellent: *Avoids medical jargon or readily explains it *Thoughts are consistently well organized and easy to understand *Uses warm and accepting tone of speech |
| 8.  Non-verbal Communication | ( )  Poor: *Unable to make eye contact *Awkward physical distance, facial expressions, or touching *Conveys disinterest or apathy | ( )  Fair | ( )  Adequate: *Maintains some eye contact *Appropriate physical distance, facial expressions, or touching *Conveys interest and concern | ( )  Very Good | ( )  Excellent: *Consistent eye contact *Uses physical distance, facial expressions, or touching effectively *Conveys attentiveness and compassion |
| 9.  Empathy | ( )  Poor: *Ignores or fails to detect emotional cues *Empathetic responses/emotional support absent or forced *Dismissive of pain or anxiety | ( )  Fair | ( )  Adequate: *Responds to emotional cues *Provides empathetic responses/emotional support *Acknowledges pain or anxiety | ( )  Very Good | ( )  Excellent: *Perceptive of emotional cues and encourages emotional expression *Provides empathetic responses/emotional support with genuineness and sincerity *Attentive to pain or anxiety |
| 10.  Respect | ( )  Poor: *Judgmental attitude *Makes you feel inferior *Physical exam without regard to pain or modesty | ( )  Fair | ( )  Adequate: *Non-judgmental attitude *Treats you as equal *Physical exam respectful of pain and modesty | ( )  Very Good | ( )  Excellent: *Accepting attitude *Establishes partnership *Physical exam with great sensitivity to pain and modesty |
| 11.  Closure | ( )  Poor: *No explanation of impression or plan *No inquiry into remaining questions *No cordial closing remarks | ( )  Fair | ( )  Adequate: *Explains impression and plan *Inquires about remaining questions *Cordial closing remarks | ( )  Very Good | ( )  Excellent: *Thorough discussion of impression and plan *Seeks unanswered questions, verification of understanding, and comfort level *Warm and grateful closing remarks |

**Comments**

| 12.  Student Feedback: |
| --- |
