# Supplementary material for: Understanding Sexual Complaints and History Taking: A Standardized Patient Case on Dyspareunia for Obstetrics and Gynecology Clerkship Students
Source: MedEdPORTAL. 2020 Oct 29;16:11001. doi: 10.15766/mep_2374-8265.11001 (PMC7597941; doi:10.15766/mep_2374-8265.11001)
Supplement: Supplementary file 1 — Preencounter SP Information.docxPreencounter Learner Information.docxPostencounter Learner Note.docxPostencounter SP Evaluation.docxPostencounter Learner Evaluation.docxPostencounter Learner Observation.docxSummary Didactic Session.docx [file mep_2374-8265.11001-s001.zip › E. Postencounter Learner Evaluation.docx]

Standardized Patient CPX Student Scoring Criteria: Dyspareunia

**Postencounter Learner Evaluation**

|  | Poor | Fair | Adequate | Very Good | Excellent |
| --- | --- | --- | --- | --- | --- |
| 1. Overall, I created an environment where the patient felt safe talking about her sexual experiences | ( ) | ( ) | ( ) | ( ) | ( ) |
| 2. My non-verbal (body language, mannerisms etc) created a comfortable environment for the patient to talk openly about her concerns regarding her sexual experiences | ( ) | ( ) | ( ) | ( ) | ( ) |
| 3.I asked questions in a way that created a comfortable environment for the patient to talk openly about her concerns regarding her sexual experiences | ( ) | ( ) | ( ) | ( ) | ( ) |
