# Supplementary material for: Understanding Sexual Complaints and History Taking: A Standardized Patient Case on Dyspareunia for Obstetrics and Gynecology Clerkship Students
Source: MedEdPORTAL. 2020 Oct 29;16:11001. doi: 10.15766/mep_2374-8265.11001 (PMC7597941; doi:10.15766/mep_2374-8265.11001)
Supplement: Supplementary file 1 — Preencounter SP Information.docxPreencounter Learner Information.docxPostencounter Learner Note.docxPostencounter SP Evaluation.docxPostencounter Learner Evaluation.docxPostencounter Learner Observation.docxSummary Didactic Session.docx [file mep_2374-8265.11001-s001.zip › F. Postencounter Learner Observation.docx]

Standardized Patient CPX Student Scoring Criteria: Dyspareunia

**Postencounter Learner Observation**

SEXUAL HISTORY: After watching your encounter with the patient, please mark below which of the following aspects of her sexual history you elicited during the encounter.

**Scoring (1 point for each)**

- Sexually active with males only
- Sexual debut at age 17
- 5 lifetime partners
- Been with current partner for 4 years (exclusive)
- No history of anal sex
- Performs oral sex, occasionally receives oral sex
- Has had orgasm in the past
- Orgasms with <20% of sexual encounters
- Uses vibrator that helps with orgasm, has never used dildo
- Sometimes masturbates
- Does not feel like adequately lubricated, uses lubricants sometimes that seem to help
- Denies issues with libido, describes it as “average”
- Denies emotional, physical or sexual abuse

**Total Points: ___/13**
