# Supplementary material for: Understanding Sexual Complaints and History Taking: A Standardized Patient Case on Dyspareunia for Obstetrics and Gynecology Clerkship Students
Source: MedEdPORTAL. 2020 Oct 29;16:11001. doi: 10.15766/mep_2374-8265.11001 (PMC7597941; doi:10.15766/mep_2374-8265.11001)
Supplement: Supplementary file 1 — Preencounter SP Information.docxPreencounter Learner Information.docxPostencounter Learner Note.docxPostencounter SP Evaluation.docxPostencounter Learner Evaluation.docxPostencounter Learner Observation.docxSummary Didactic Session.docx [file mep_2374-8265.11001-s001.zip › G. Summary Didactic Session.docx]

**Standardized Patient CPX Student Encounter: Dyspareunia**

**Summary Didactic Session**

*Objectives:*

1. Identify the key communication skills and recommended questions for a thorough sexual history.
2. Outline the signs, symptoms and physical exam and laboratory findings associated with the differential diagnosis for dyspareunia.
3. List the initial treatment options for women presenting with dyspareunia.

*Case Presentation:*

A 25-year-old G1P1001 presents to your gynecology clinic for an annual exam. She had a vaginal delivery 6 months ago and is still breastfeeding. On review of systems, she notes that she has tried to resume intercourse with her male partner since delivery but has significant pain that prevents her from having intercourse. She notes that she previously had mild discomfort while having sex, but it has worsened since giving birth. She previously had both oral and vaginal intercourse with this partner and was able to achieve orgasm occasionally. Her only medications are Citalopram, which she was prescribed by her obstetrician for postpartum depression, and the patch, which she started 6 weeks postpartum. On examination, her heart rate is 72 beats per minute, her blood pressure is 119/69, and her temperature is 98.4 F (36.9 C). Pelvic exam is notable for tenderness to palpation with a cotton swab around the vaginal introitus, evidence of mild vaginal atrophy, and a slightly malodorous discharge in the vaginal vault.

**🡪 What is the differential diagnosis?**

**🡪 What options does the patient have for treatment?**

Answers:

*Summary:* A 25-year-old G1P1001 complains of dyspareunia 6 months following vaginal delivery. She has normal vital signs, but her exam is notable for vulvar pain, vaginal atrophy, and new vaginal discharge.

- **Differential Diagnosis:**
  - Vaginismus
  - Vulvodynia
  - Vulvovaginitis
  - Medication side effect
  - Decreased genital arousal (including due to vaginal atrophy)
  - Birth trauma
- **Treatment options:**
  - Continued lubricant use
  - Discussion of foreplay and other arousal techniques
  - Referral to pelvic floor physical therapy
  - Referral to sex therapy
  - Discussion of changing anti-depressant medication
  - Discussion of changing contraception

*Clinical Approach:*

Sexual pain is common among women, with 8 to 21% of women globally reporting symptoms [1]. Dyspareunia is defined as recurrent or persistent genital pain associated with sexual intercourse that causes marked distress or interpersonal difficulty [2]. Conditions associated with sexual pain can be characterized by either superficial or deep pain. While only some causes of sexual pain are discussed below, a full differential diagnosis is presented in figure 1 [2,3].

**Figure 1. Conditions Associated with Sexual Pain.**

| Superficial Sexual Pain | Deep Sexual Pain |
| --- | --- |
| *Vulvar Pathology* | *Uterine Pathology* |
| Vulvar Dermatoses (Lichen Planus, Lichen Sclerosis, Lichen Simplex Chronicus) | Adenomyosis |
| Vestibulodynia/Vulvodynia | Uterine leiomyomas |
| Vulvar condylomas | *Adnexal Pathology* |
| *Vaginal Pathology* | Endometriosis |
| Vaginismus | Pelvic inflammatory disease |
| Vulvovaginitis/Vaginitis | Pelvic congestion syndrome |
| Levator spasm | *Surrounding Organ Pathology* |
| Decreased lubrication/Medication effect | Irritable bowel syndrome |
| Obstetric complications/Birth trauma | Interstitial Cystitis |

*Sexual History*

One of the most important aspects of determining the etiology of sexual pain is eliciting an appropriate sexual history. Asking open ended questions and normalizing patient responses can be key elements in obtaining accurate information from patients. Questions on sexual activity may include the following [4]:

1. Sexual partners: Is the patient currently sexually active? How many sexual partners have they had? Are their sexual partners men, women, or both?
2. Sexual practices: What sexual practices is the patient engaging in (oral, vaginal, or anal intercourse)? Are there other ways they are intimate with their partner?
3. Sexual satisfaction: Is the patient satisfied with her sexual interactions? Does she think her partner is satisfied?
4. Sexual function: Does the patient have orgasms? Does she feel like she has adequate vaginal lubrication?
5. Protection from STIs: Is the patient using anything to protect against STIs? Has she been diagnosed with an STI in the past?
6. Prevention of Pregnancy: Is the patient using anything for birth control? Is she interested in becoming pregnant in the next year?

A sexual history should be followed by a medical, surgical, social, and psychiatric history. Underlying anxiety disorders, as well as discord with an intimate partner or intimate partner violence can increase the likelihood of dyspareunia [3]. A physical exam should focus on a thorough gynecologic exam; including assessment of vulvar sensation with a cotton swab and assessment of the pelvic floor musculature. A wet prep should be performed as well.

*Vaginismus*

Vaginismus occurs in 1 to 6% of women and may present with a wide array of symptoms [2]. Vaginismus is defined as recurrent or persistent involuntary spasm of the musculature of the outer third of the vagina. Some women with vaginismus only have pain with intercourse, but others have pain with pelvic examination as well. Similarly, some women may only have pain with vaginal penetration during intercourse, while others may have pain on any genital contact. Women with vaginismus frequently also have hypoactive sexual desire disorder or sexual aversion; which may contribute to worsening physical discomfort due to the lack of genital arousal [2].

*Vulvodynia*

Vulvodynia is a diagnosis of exclusion, encompassing vulvar pain of at least 3 months duration without a clear identifiable cause [5]. Vulvar vestibular syndrome is thought of as localized vulvodynia and is the most common cause of sexual pain at the introitus [3]. A cotton swab is used during pelvic exam to determine specific areas of pain. Other diagnoses that should be ruled out prior to making this diagnosis are shown in figure 2 [5].

**Figure 2.** **Persistent vulvar pain treatment algorithm.**

*Vulvovaginitis*

Vulvovaginitis encompasses infectious and inflammatory causes of dyspareunia, and is associated with other vulvovaginal symptoms including itching, burning, irritation, and abnormal discharge [6]. The most common causes of vulvovaginitis include bacterial vaginosis, vulvovaginal candidiasis, and trichomoniasis. Other non-infectious etiologies of vaginitis include atrophic vaginitis, irritant/allergic vaginitis, and inflammatory vaginitis. Differentiation of these causes include a wet prep, potassium hydroxide prep, and determination of vaginal pH. Symptoms relating to these causes of vulvovaginitis are shown in figure 3 [7].

**Figure 3. Signs and Symptoms of Vaginitis.**

| Diagnosis | Etiology | Signs/Symptoms | Vaginal pH | Wet Prep Findings | Treatment |
| --- | --- | --- | --- | --- | --- |
| Bacterial vaginosis | Anaerobic bacteria (*Gardnerella vaginalis*, *Prevotella*, *Mobiluncus*, *Ureaplasma*, *Mycoplasma*) | Thin grey discharge; “fishy” odor; diffuse pelvic pain | Elevated (>4.5) | Clue cells | Metronidazole 500mg PO BID for 7-day regimen |
| Vulvovaginal candidiasis | *Candida albicans*, occasionally other *Candida* species | White, thick, cheesy discharge; vulvar itching or burning | Normal | Pseudohyphae, budding yeast | Fluconazole 150mg PO once |
| Trichomoniasis | *Trichomonas vaginalis* | Yellow-green, frothy discharge; foul odor; strawberry cervix; vaginal pain | Elevated (>4.5) | Trichomonads | Metronidazole 2g PO once |
| Atrophic vaginitis | Estrogen deficiency | Thin, clear discharge; vaginal dryness and itching; thin, friable vaginal mucosa | Elevated (>5) | Increased parabasal cells | Vaginal estrogen twice weekly |
| Irritant/allergic vaginitis | Contact irritation or allergic reaction | Vulvar erythema; burning, soreness | -- | -- | Vulvar hygiene measures; remove irritant |
| Inflammatory vaginitis | Possibly autoimmune | Purulent vaginal discharge; vaginal atrophy; vaginal inflammation; burning | -- | -- | Vulvar hygiene measures; possible steroids |

*Medication Side Effect*

Medications that have been associated with female sexual dysfunction include psychotropic medications, antihypertensives, histamine blockers, and hormonal medications. Among psychotropic medications, SSRIs may cause decreased sexual desire, arousal, and orgasmic dysfunction [2]. Low-dose estrogen oral contraceptive pills can also be associated with decreased genital arousal and lubrication [3].

*Decreased Genital Arousal*

Vaginal lubrication is a product of the vaginal wall epithelium and relies on the vascular supply to the epithelium as well as estrogen. Female sexual arousal disorder is the inability to complete sexual activity with adequate lubrication that causes distress [2]. However, decreased genital arousal can also have secondary causes such as discord with an intimate partner, medication effects (as above), or hypoestrogenic effects.

*Birth Trauma*

Changes in sexual health affect more than half of women after childbirth, and can include lack of interest, lack of arousal, and dyspareunia. Obstetric lacerations are associated with dyspareunia, with women experiencing higher degree lacerations or episiotomies are more likely to have pain with intercourse than those without lacerations [3]. Postpartum depression can also decrease interest, arousal, and orgasm during sexual encounters [9]. For women who are breastfeeding, elevated prolactin decreases ovarian production of androgens and estrogens, decreasing arousal and vaginal lubrication [9]. Even after a workup for known causes of dyspareunia following childbirth, approximately 24% of women have idiopathic dyspareunia 6 months after delivery, which decreases without treatment to about 8% at 1 year after delivery [3]. Discussion of sexual health is an important part of pre-conception counseling and postpartum visits.

*Treatment Options*

Treatment options for dyspareunia should be targeted towards the underlying cause of the patient’s pain. For those with decreased genital arousal, lubrication use, particularly with a water-based lubricant, can help in decreasing symptoms of vaginal dryness. For vaginal dryness related to atrophy in the menopausal population, vaginal estrogen applied twice weekly may also improve vaginal lubrication. Vulvodynia or other pain with a possible neuropathic pain response may benefit from other local therapy including topical local anesthetics or topical compounded tricyclic antidepressants [5]. Pelvic floor physical therapy has been shown to be successful for dyspareunia, particularly for patients with a muscular component [3]. This physical therapy may involve electrical stimulation or biofeedback to aid patients in identifying when and how they use their pelvic floor muscles to help decrease muscle spasm. Sex therapy can be helpful for women with a wide range of challenges related to sexual function. For patients using medications that may lead to decreased arousal or lubrication, discussion of changing those medications may result in improved symptoms. Many women have multiple causes for their dyspareunia and relapse rates following treatment can be high; so continued discussion and therapy should be seen as a vital part of a routine gynecologic visit.

*Comprehension Questions:*

1. A 38-year-old woman presents to your clinic complaining of pain on intercourse. Her past medical history is notable for anxiety/depression, hypothyroidism, and diabetes. Which of the following medications that she is taking may cause the most sexual side effects?
   1. Levothyroxine
   2. Fluoxetine
   3. Metformin
   4. Alprazolam
2. A 25-year old woman presents to your clinic complaining of increased pain during intercourse that has been accompanied by increased vaginal discharge with a fishy odor. You perform a pelvic exam and obtain a wet prep where you see clue cells. What is the most appropriate therapy?
   1. Clindamycin
   2. Azithromycin
   3. Metronidazole
   4. Doxycycline
3. A 29-year old woman comes to your office due to sexual pain that has been distressing to her and her partner. On physical exam, you note spasms when you palpate her pelvic floor muscles, which she says reproduces the pain that she notices during intercourse. Which of the following may be the most appropriate first line therapy?
   1. Referral to pelvic floor physical therapy
   2. Reassure patient that this is a normal response, and she just has to get used to the pain
   3. Prescription of topical local anesthetic
   4. Referral to sex therapy
4. A 32-year old woman comes to your office for an annual gynecologic exam. Which topic should be included in your discussion of her sexual history?
   1. Number and sex of sexual partners
   2. Protection against STIs and pregnancy
   3. Sexual function including lubrication and ability to orgasm
   4. All of the above

*References:*

1. Latthe P, Latthe M, Say L et al. 2006. WHO systematic review of prevalence of chronic pelvic pain: a neglected reproductive health morbidity. BMC Public Health; 6: 177.
2. ACOG. 2011. Practice Bulletin 119: Female Sexual Dysfunction.
3. Steege JF, Zolnoun DA. 2009. Evaluation and Treatment of Dyspareunia. Obstet Gynecol; 113: 1124-36.
4. ACOG. 2017. Committee Opinion 706: Sexual Health.
5. ACOG, ASCCP. 2016. Committee Opinion 673: Persistent Vulvar Pain.
6. ACOG. 2006. Practice Bulletin 72: Vaginitis.
7. Paladine HL, Desai UA. 2018. Vaginitis: Diagnosis and Treatment. Am Fam Physician; 97(5): 321-9.
8. O’Malley D, Higgins A, Begley C et al. 2018. Prevalence of and risk factors associated with sexual health issues in primiparous women at 6 and 12 months postpartum: a longitudinal prospective cohort study (the MAMMI study). BMC Pregnancy and Childbirth; 18:196.
9. Leeman LM, Rogers RG. 2012. Sex After Childbirth: Postpartum Sexual Function. Obstet Gynecol; 119: 647-55.

*Comprehension Question Answers:*

1. B
2. C
3. A
4. D
